# MTBP enhances the activation of transcription factor ETS-1 and promotes the proliferation of hepatocellular carcinoma cells

**DOI:** 10.3389/fonc.2022.985082

**Published:** 2022-08-29

**Authors:** Hongbo Wang, Fang Chu, Li Zhijie, Qian Bi, Li Lixin, Yunlong Zhuang, Zhang Xiaofeng, Xiaofeng Niu, Dali Zhang, He Xi, Bo-an Li

**Affiliations:** ^1^ Senior Department of Hepatology, The Fifth Medical Center of PLA General Hospital, Beijing, China; ^2^ Department of Emergency, The Fifth Medical Center of Chinese People’s Liberation Army General Hospital, Beijing, China; ^3^ Endoscopy Center, Department of Hepatology, The Fifth Medical Center of Chinese People’s Liberation Army General Hospital, Beijing, China; ^4^ Clinical Laboratory, The Fifth Medical Center of Chinese People’s Liberation Army General Hospital, Beijing, China

**Keywords:** Hepatocellular carcinoma, MDM2 binding protein, E26 transformation specific sequence 1, proliferation, transcription factor activation, protein interaction

## Abstract

Increasing evidence indicates that the oncoprotein murine double minute (MDM2) binding protein (MTBP) can be considered a pro-oncogene of human malignancies; however, its function and mechanisms in hepatocellular carcinoma (HCC) are still not clear. In the present work, our results demonstrate that MTBP could function as a co-activator of transcription factor E26 transformation-specific sequence (ETS-1), which plays an important role in HCC cell proliferation and/or metastasis and promotes proliferation of HCC cells. Using luciferase and real-time polymerase chain reaction (qPCR) assays, MTBP was found to enhance the transcription factor activation of ETS-1. The results from chromatin co-immunoprecipitation showed that MTBP enhanced the recruitment of ETS-1 to its downstream gene’s (mmp1’s) promoter region with ETS-1 binding sites. In cellular and nude mice models, overexpression of MTBP was shown to promote the proliferation of MHCC97-L cells with low endogenous MTBP levels, whereas the knockdown of MTBP led to inhibition of the proliferation of MHCC97-H cells that possessed high endogenous levels of MTBP. The effect of MTBP on ETS-1 was confirmed in the clinical specimens; the expression of MTBP was positively correlated with the downstream genes of ETS-1, *mmp3*, *mmp9*, and *uPA*. Therefore, by establishing the role of MTBP as a novel co-activator of ETS-1, this work expands our knowledge of MTBP or ETS-1 and helps to provide new ideas concerning HCC-related research.

## Introduction

Hepatocellular carcinoma (HCC) is one of the most fatal and common malignancies in China due to the high rates of hepatitis virus infection (hepatitis B and C viruses [HBV and HCV, respectively]) (1–4). Therefore, elucidation of the molecular mechanisms related to the tumorigenesis, metastasis, and/or progression of HCC is important and valuable for HCC treatment (5, 6). Although it is well known that HCC is related to aberrant activation of receptor tyrosine kinases (RTKs) or RTK-activated intracellular cascades (mitogen-activated protein kinase [MAPK] or phosphoinositide-3-kinase/protein kinase B [PI3K/AKT]), the genetic alterations related to the tumorigenesis, metastasis, and/or progression of HCC remains poorly understood and the in-depth research work is a necessity (7–10). Transcription factor E26 transformation specific sequence 1 (ETS-1), which belongs to the ETS transfection factor family that is structurally featured as the ETS domain, mediates proliferation, development, metastasis, and/or angiogenesis of multi-kinds of human cancerous cells by binding to the ETS-binding elements (EBS motif; 5’-GGAA/T-3’ sequences), located in the promoter or enhancer of its downstream genes (11–13). The aberrant expression or activation of ETS-1 is associated with poor prognosis of human malignancies (14, 15); thus, ETS-1 is also a potential therapeutic target and it is valuable to further explore the roles and mechanisms of ETS-1 in HCC.

MTBP (MDM2 binding protein) has been regarded as a novel pro-oncogene of human cancer cells (16, 17). Some publications revealed that MTBP could promote the proliferation or metastasis of human cancerous cells, however, the detailed mechanism of MTBP is still not clear (16, 17). A promising mechanism of MTBP-associated function is to act as the co-activator of pro-oncogenes, such as cellular-myelocytomatosis viral oncogene (c-Myc) or Zinc Finger E-Box Binding Homeobox 2m (ZEB2) (17–20). Our previous results showed that MTBP was found to act as a co-activator of the Pregnane X receptor (PXR), a nuclear receptor/transcription factor in HCC cells (21). However, the association between MTBP with HCC remains unclear. In this study, our results showed that MTBP functioned as a novel co-activator of ETS-1 and promoted HCC cellular proliferation *via* enhancement of ETS-1 activation.

## Materials and methods

### cDNA samples from HCC clinical specimens and hepatic cell lines

The cDNA samples from a total number of 52 patients with advanced HCC were described in our previous publications and frozen at −80°C (22, 23). The hepatic cell line, MHCC97-H (a highly aggressive HCC cell line), MHCC97-L (a low aggressive HCC cell line) or Huh-7 cells (a HCC cell line with deficient P53) and HEK293 cells were prepared in our lab and described in our previous work (22, 23). In cellular experiments, 10% (v/v) fetal bovine serum (FBS, Invitrogen, Thermo Fisher Scientific Corporation, Waltham, MA, USA) in Dulbecco’s Modified Eagle Medium (DMEM, Hyclone, Thermo Fisher Scientific Corporation, Waltham, MA, USA) was added to cell cultures at 37°C in 5% CO_2_. The inhibitor of the hepatocyte growth factor (HGF)/c-MET/ETS-1 pathway, ARQ-197 (Cat No.: S2753) (24), was purchased from Selleck Corporation, Houston, TX, USA. The pure powder of ARQ-197 was formulated into a drug solution according to the method reported in the publications/references (25–28). The agonist of the c-MET/ETS-1 pathway, HGF, was purchased from Pepro-Tech Corporation, Rocky Hill, NJ, USA. HGF was diluted phosphate-buffered saline (PBS) and stored at −80 °C.

The expression vectors containing full length sequences of MTBP or siMTBP for cellular experiments were described in our previous publication (21). For animal experiments, the full-length sequences of MTBP or MTBP siRNA were prepared as lentivirus particles (Vigene Corporation, Jinan City, Shandong Province, China). The HCC cells were cultured and stably transfected (MHCC97-H cells for MTBP knockdown and MHCC97-L cells for MTBP overexpression) with the lentivirus particles according to the instruction obtained from the manufacture (Vigene Corporation). The HCC cells were cultured and counted (using a fully automated cell counter), and approximately 10^9^ pfu amount of lentivirus was added to about 5 × 10^6^ amount of HCC cells, followed by the screening for stable transfection (the MTBP overexpression was screened by using the G418, and MTBP knockdown was screened by using the puromycin).

### Luciferase assay

The activity of ETS-1 in HCC cells and the effect of MTBP on ETS-1 were measured using the luciferase assay. Detection of ETS-1 downstream gene reporter activity was done after MTBP overexpression or MTBP knockdown in HCC cells. In the present work, luciferase reporters included EBS-Luc (a gift from Professor and Dr. Yinjie Gao in the Beijing 302^nd^ hospital) or MMP1-Luc, uPA-Luc (gifts from Professor and Dr. Fan Zhang in the General Hospital of Chinese PLA) (29, 30). These luciferase reporters were prepared as: the promoter region sequence containing the binding sites of *mmp1* or *uPA* was cloned into the pGL3-Basic plasmids. For luciferase reporter of EBS-Luc, the octamerEBS (ETS binding site) sequence (GGAA)_8_ was synthesized by using chemical synthesis methods and cloned into the pGL3-Promoter plasmids (29, 30).

The HCC cells (MHCC97-H, MHCC97-L, HEK293 cells or Huh-7) transfected with plasmids (MTBP or siMTBP co-transfected with luciferase reporters) were prepared as a single cell suspension and then seeded onto 24-well cell culture plates (Corning, Corning, NY, USA) in phenol red-free DMEM (Gibco Corporation, Grand Island, NY, USA) with 0.5% charcoal-stripped FBS (Hyclone, Logan, UT, USA) with or without 20 ng/ml HGF. After 34 h, luciferase and β-galactosidase activities were examined according to previously described methods (31, 32). The results are shown as histogram based on relative luciferase activation (fold of control).

### Quantitative polymerase chain reaction

The expression of *MTBP*, *mmp3*, *mmp9*, and/or *uPA* in the cDNA of the patients was directly examined by quantitative polymerase chain reaction (qPCR; the one-step real-time PCR). For cellular experiments, the cells were cultured and transfected with plasmids. These mRNA samples were then extracted from the HCC cells and reverse-transcribed into cDNA. This reverse transcription was performed by RNeasy Mini kit (Qiagen, Valencia, CA, USA) according to the manufacturer’s instructions (Qiagen, Valencia, CA, USA). The qPCR experiments were performed according to the methods provided in references (33, 34). The endogenous level of β-actin’s mRNA was measured and used as a loading control to determine the relative expression level of genes’ mRNA. The sequences of primers used in the qPCR experiments are listed: (1) MTBP forward sequences 5′-TCCTGTAGTTTCGTCAGATCCT-3′ and reverse sequences 5′-CCGTTTCAATCGGGATACTTCA-3′, (2) MMP-3 Forward sequences 5’-CACTCACAGACCTGACTCGGTT-3’ and Reverse sequences 5’-AAGCAGGATCACAGTTGGCTGG-3’, (3) MMP-9 Forward sequences 5’-GCCACTACTGTGCCTTTGAGTC-3’, Reverse sequences 5’-CCCTCAGAGAATCGCCAGTACT-3’, (4) uPA/PLAU Forward sequences 5’-TTGCTCACCACAACGACATT-3’ and Reverse sequences 5’-ATTTTCAGCTGTCCGGATA-3’, and (5) loading control β-actin forward sequences 5’-CACCA TTGGCAATGAGCGGTTC-3’ and reverse sequences 5’-AGGTCTTTGCGGATGTCC ACGT-3’.

### The co-immunoprecipitation and western-blot

For co-immunoprecipitation experiments, MHCC97-H cells were transfected with FLAG-MTBP vector. After that, the cells were disrupted by sonication, centrifuged at 12000 rpm at 4°C for about 5 min, and the supernatant was separated to obtain a cell sample. After that, use high-salt IP buffer to mix well with the supernatant of the previous step and beads coupled with FLAG-tagged monoclonal antibody (FLAG-beads, stored in this laboratory, 4°C), and then rotate on a low-speed rotary shaker. Incubate overnight at 4°C. After enrichment is completed, centrifuge at 800 rpm for 3 min at 4°C to separate the FLAG-Beads, that is, to complete the co-immunoprecipitation. At this time, FLAG-MTBP and ETS-1 are bound to FLAG-beads (that is, a ternary complex of FLAG-MTBP, FLAG-beads and ETS-1 is formed. In this step, such a ternary complex/separated out is removed from the system). The isolated FLAG-beads were further analyzed by western blot.

The protein samples from the FLAG-beads or extracted from HCC cells were analyzed by western blot experiments. For FLAG-beads, the beads were directly subjected to a boiling water bath for 15 minutes, and then centrifuged at 12,000 rpm for about 5 minutes, and the supernatant was collected as the protein sample. For cell samples, use a plastic scraper to detach the cells from the surface of the dish or flask, and then collect the cells and wash them gently with PBS 2-3 times (each time gently mix the cells with PBS and centrifuge at 800rpm, 4°C) About 5min is washed once), then take a boiling water bath for 15-20min, and finally centrifuge at 4°C for about 5-10min, collect the supernatant as a protein sample. After that, the two protein samples were subjected to SDS-PAGE, membrane transfer, blocking, and antibody incubation, respectively, following the methods described in our previous publications. During these steps, the antibodies against FLAG or ETS-1 were purchased from Abcam Corporation, Cambridge, UK. The blots/membranes were visualized *via* the chemiluminescence by use of an ECL kit (Amersham Biosciences, Piscataway, NJ, USA) and the images of western blot were quantitatively analyzed by Image J software.

### The ChIP experiments

Chromatin co-immunoprecipitation ChIP detection was performed after transfection of the corresponding vector in HCC cells. The MTBP was overexpressed in MHCC97-L cells and MTBP was knocked down using its siRNA in MHCC97-H cells. After transfection, the cells were treated with 20 ng/ml rifampicin for 30 min. Cells were then harvested for ChIP analysis and the experiments were performed following the methods described by Ma et al. and Wang et al. (35, 36). The primers used to amplify the promoter region (the PXRE region [−362/+52]) of the promoter of cyp3a4 included several sets: (1) forward primer: 5’-AGATCTGTAGGTGTGGCTTGTTGG-3’, (2) reverse primer: 5’-TGTTG CTCTTTGCTGGGCTA TGTGC-3’, (3) Input (genome DNA sequence): forward primer: 5’-AA CCTATTAACTCACCCTTGT-3’, and (4) reverse primer: 5’-CCTCCATTCAAAAGATCTTATTATTTAG CATCTCCT-3’. Recruitment of ETS-1 to its downstream gene’s promoter was revealed by the relative recruitment (fold-value of the input) (37).

### The MTT and transwell experiments

The *in vitro* proliferation, invasion or migration was examined by the MTT and transwell experiments. For MTT experiments, HCC cells (including MHCC97-H, MHCC97-L, and Huh-7 cells) were cultured, transfected, and then seeded in 96-well cell culture plates (about 2000-3000 cells per well) (38, 39). Then cells were cultured in DMEM supplemented with 10% FBS, and the MTT experiment was performed after 48 h. The general procedure of MTT experiment is: add MTT reagent (50mmol/L dose) to 96 wells seeded with cells. After 5-6 h of incubation, the entire liquid in the wells was discarded, and the cell samples were finally lysed with DMSO. During this process, the 96-well plate was shaken by a vortex shaker, and finally the plate was centrifuged, and the supernatant sample was collected to detect the absorbance value at 490nm wavelength (O.D. 490nm).

For Transwell experiments (40, 41), HCC cells were prepared as a cell suspension, and then added the cell-suspension into the upper chambers of the Transwell plates. At this time point, DMEM containing 10% FBS was added to the lower layer of the Transwell plates, and then the chamber was placed in the lower layer and incubated at 37°C. For *in vitro* invasion experiments, the ECM at the bottom of the chamber was added before the cell suspension; for *in vitro* migration experiments, the cell suspension was directed added into the the bottom of the chamber. The Tanswell plates were incubated at 37°C (14-16h for invasion transwell; 6-8h for migration transwell). After incubation, the Chambers were fixed with methanol, and then stained with 0.5% (v/v diluted by 70% ethanol) for about 15 min. After staining, use a cotton swab to gently wipe off non-transferred or invaded cells. After washing the chambers three times with deionized water, and finally soaking the outside of the chambers with absolute ethanol to extract the crystal violet in the cells, read the absorbance value using a full-wavelength multi-function microplate reader at a wavelength of 546nm.

### *In vivo* HCC tumor model

The effect of MTBP on the proliferation of HCC cells *in vivo*, especially the activity of ETS-1, was detected using a nude mouse tumorigenic model (42, 43). MHCC97-H, MHCC97-L cells or Huh-7 cells were cultured, and siMTBP was stably transfected in MHCC97-H cells, the expression vector of MTBP was stably transfected into MHCC97-L cells or the siRNA of MTBP/expression vector of MTBP was stably transfected into Huh-7 cells after which the cells were collected to prepare the cells as a single cell suspension. The suspension was injected subcutaneously into nude mice (the specific location was near the inner groin vein of the lower limbs of nude mice). After the injection was completed, the nude mice were continuously reared under specific pathogen free (SPF) conditions, and the status of the mice such as whether they survived and/or whether any skin ulcerations had occurred, was observed every other day.

After continuous feeding, HCC cells formed tumor tissues in nude mice (MHCC97-L cells, took 8-10 weeks to grow; MHCC97-H cells took about 6 weeks; Huh-7 cells took about 8 weeks). After continuous feeding, HCC cells were able to form tumor tissue in nude mice (MHCC97-L cells took 8–10 weeks to grow; MHCC97-H cells took about 6 weeks; Huh-7 cells took about 8 weeks). Thereafter, tumor tissue was collected, and the weights and volumes of the tumor tissue were determined. Finally, the tumor tissue was ground in liquid nitrogen, and then total RNA samples were extracted for reverse transcription and qPCR detection (44, 45). The expression levels of mmp3 and other related factors were detected in the tumor tissues of each group, and the results were shown as histograms.

### The ethics statement

The use of clinical specimen’s related materials and human resourced cell lines were approved by the ethics committee of the Fifth Medical Center, General Hospital of Chinese PLA (People’s Liberation Army). All experiments were carried out in accordance with the Helsinki Declaration policy. For animal experiments, the purchase, preservation, feeding and use of experimental animals, in addition to the complete experimental design and technology of animal experiments were approved and filed by the Animal Ethics Committee of the Fifth Medical Center of the Chinese People’s Liberation Army General Hospital.

### Statistical analysis

Statistical significance analyses were analyzed by using the statistical software (software version: SPSS 9.0, the IBM corporation, Armonk, NY, USA). The statistical significance was analyzed by Bonferroni correction with or without two-way analysis of variance (ANOVA).

## Results

### MTBP enhances the transcription factor activation of ETS-1 in HCC cells

**Figure 1:**
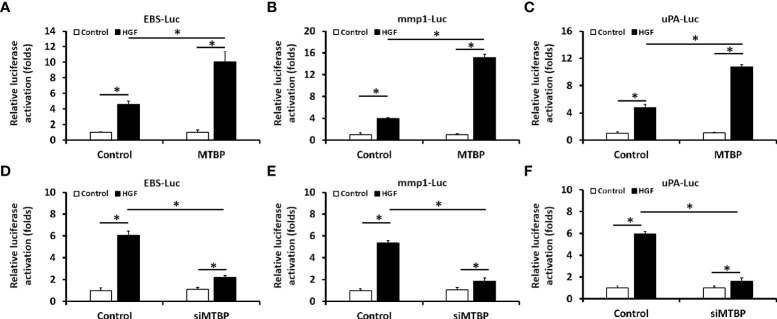
Oncoprotein murine double minute (MDM2) binding protein (MTBP) causes enhancement of the transcription factor activation of transcription factor E26 transformation specific sequence (ETS-1) in hepatocellular carcinoma (HCC) cells. **(A–C)** The effect of MTBP overexpression in MHCC97-L cells on the activation of luciferase reporters (A [ETS-Luc]; B [mmp1-Luc]; C [uPA]) induced by hepatocyte growth factor (HGF). **(D–F)** The effect of MTBP knockdown (MTBP siRNA) in MHCC97-H cells on the activation of luciferase reporters (A [EBS-Luc]; B [mmp1-Luc]; C [uPA]) induced by HGF. *P < 0.05.

**Figure 2:**
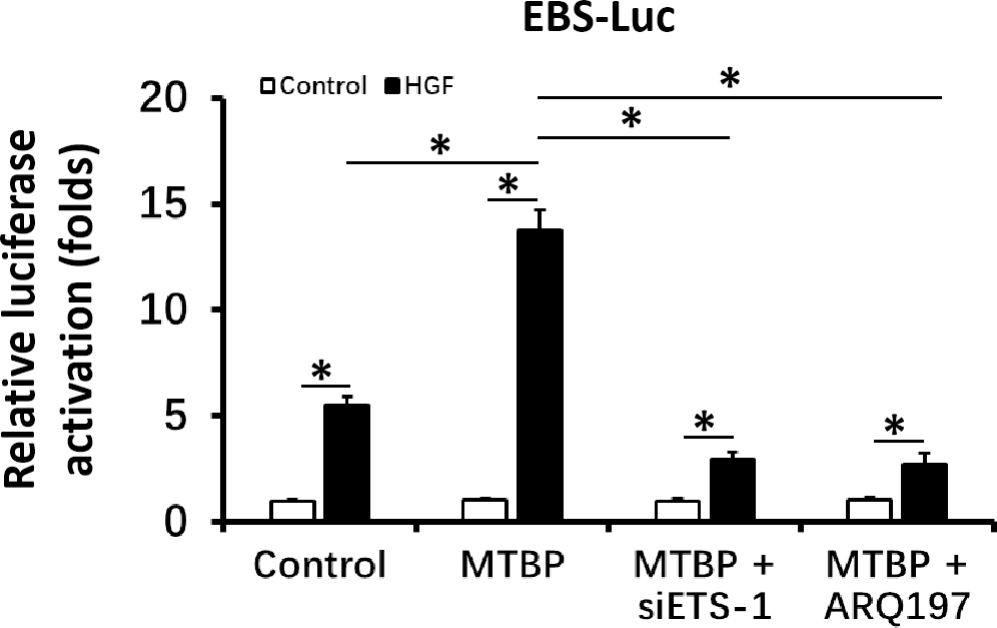
MTBP leads to enhancement of the activation of luciferase reporter EBS-Luc in an ETS-1 dependent manner in HCC cells. The effect of siETS-1 or ARQ-197 with MTBP overexpression in MHCC97-L cells on the activation of luciferase reporters (EBS-Luc) induced by HGF. *P < 0.05.

First, the effect of MTBP effects on the transcription factor activation of ETS-1 was examined by luciferase. As shown in **Figure 1**, overexpression of MTBP not only enhances the activation of EBS-Luc (**Figure 1A**) but also enhances the activation of MMP1-Luc (**Figure 1B**) and uPA-Luc (**Figure 1C**) in MHCC97-L cells. Knockdown of MTBP led to a decrease in the activation of luciferase reporters of ETS-1 in MHCC97-H cells (**Figures 1D-F**). Moreover, MTBP was found to modulate the activation of ETS-1 in the presence of HGF (in a ligand/agonist-dependent manner) (**Figure 1**). Next, the specificity of MTBP on ETS-1 was examined. As shown in **Figure 2**, knockdown of ETS-1 with its siRNA or treatment of ARQ-197 in MHCC97-L caused a decrease in the effect of MTBP overexpression on EBS-Luc’s activation. Therefore, MTBP appears to enhance the activation of luciferase reporters *via* ETS-1.

**Figure 3:**
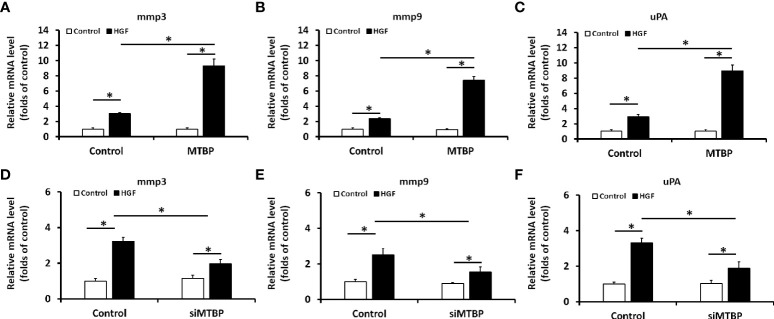
MTBP leads to enhancement of the mRNA level of ETS-1’s downstream genes in HCC cells. **(A–C)** The effect of MTBP overexpression in MHCC97-L cells on the RNA level of ETS-1’s downstream genes (A [mmp3]; B [mmp9]; C [uPA]) induced by HGF. **(D–F)** The effect of MTBP knockdown (siRNA of MTBP) in MHCC97-H cells on the RNA level ETS-1’s downstream genes (A [mmp3]; B [mmp9]; C [uPA]) induced by HGF. *P < 0.05.

The above results were obtained from luciferase activation, and qPCR was performed to further examine the effect of MTBP on ETS-1. As shown in **Figures 3A–C**, overexpression of MTBP led to enhancement of the mRNA level of *mmp3*, *mmp9*, or *uPA*, three typical downstream genes of ETS-1, in the presence of HGF; whereas knockdown of MTBP caused a decrease in the mRNA level of *mmp3*, *mmp9*, or *uPA* in the presence of HGF (**Figures 3D–F**). These results indicate that MTBP lead to enhancement of the transcription factor activation of ETS-1 in HCC cells.

### The expression level of MTBP positively related to the expression of ETS-1’s downstream genes in HCC clinical specimens

**Figure 4:**
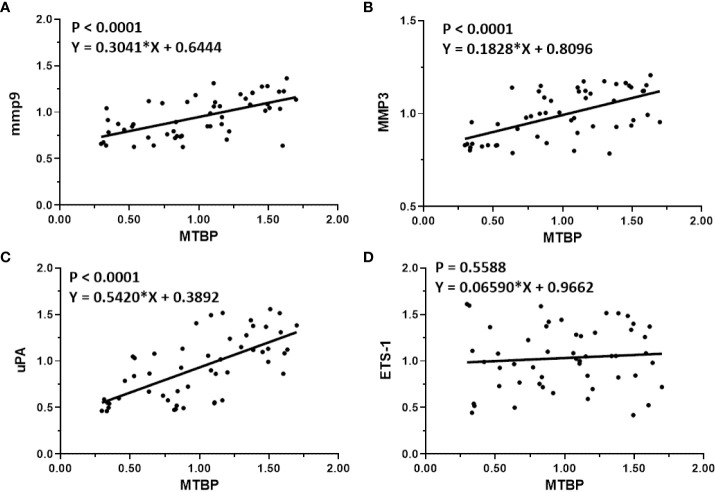
Co-relations between MTBP with ETS-1’s downstream genes in HCC clinical specimens. The scatter plot was drawn with the expression level of MTBP as the abscissa and the expression levels of mmp9 **(A)**, mmp3 **(B)**, uPA **(C)**, and ETS-1 **(D)** as the ordinate. The results also show the P-value and regression equation of the linear regression fitting results.

The previous results indicate that MTBP can act as a co-activator of ETS-1 and then upregulate the expression levels of downstream genes of ETS-1. This finding was further validated in clinical specimens. The results are shown in **Figure 4**. In HCC tumor tissues, MTBP positively correlated with the downstream genes of ETS-1, mmp9 (**Figure 4A**), mmp3 (**Figure 4B**), or uPA (**Figure 4C**) in the HCC clinical specimens and the regression equation and P values of **Figures 4A-C** were P < 0.0001, Y = 0.3041*X + 0.6444; P < 0.0001, Y = 0.1828*X + 0.8096; P < 0.0001, Y = 0.5420*X + 0.3892; respectively. However, MTBP was not correlated with ETS-1 expression (P = 0.5588, Y = 0.06590*X + 0.9662). This result further confirms the specificity of MTBP actions on ETS-1.

### MTBP interacts with ETS-1 in the presence of HGF

**Figure 5:**
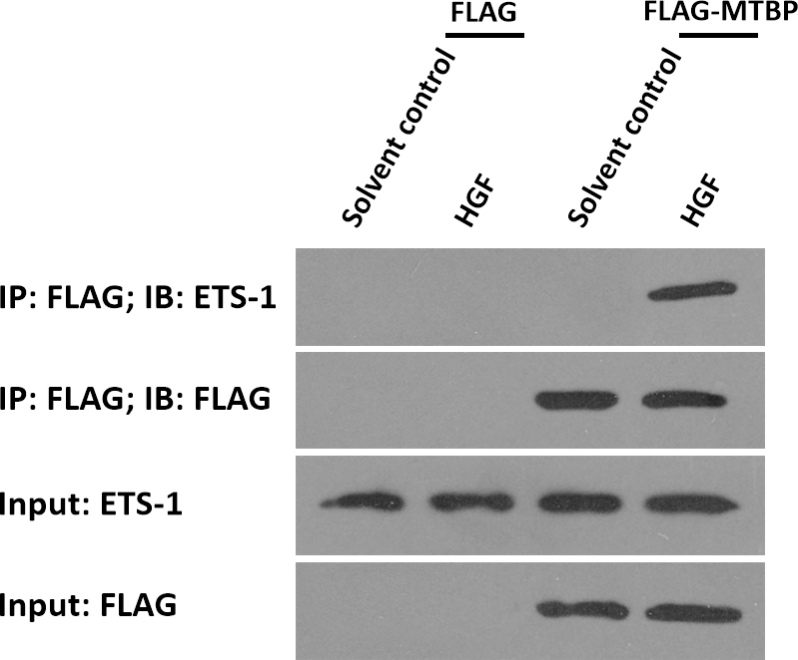
MTBP interacts with ETS-1 in MHCC97-H cells. MHCC97-H cells were cultured and transfected with FLAG vectors or FLAG-MTBP vectors. Then, MHCC97-H cells were harvested for co-immunoprecipitation assays in the absence or presence of HGF. The interaction between MTBP or ETS-1 was shown as “IP: FLAG; IB: ETS-1”.

To further examine the effect of MTBP on ETS-1, the potential protein-protein interaction between MTBP with ETS-1 was examined by co-immunoprecipitation. As shown in **Figure 5**, FLAG-MTBP could interact with ETS-1 in MHCC97-H cells in the presence of HGF. Therefore, MTBP interacts with ETS-1 in the presence of HGF.

### MTBP enhances the recruitment of ETS-1 to its downstream gene’s promoter region in the presence of HGF

**Figure 6:**
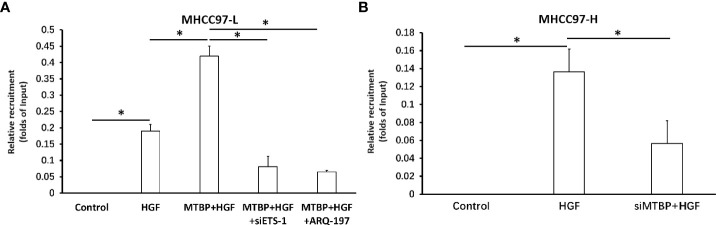
Effects of MTBP on the recruitment of ETS-1 to its downstream gene mmp1’s promoter region. **(A)** The effect of MTBP overexpression in MHCC97-L cells on the on the recruitment of ETS-1 to its downstream gene mmp1’s promoter region induced by HGF. **(B)** The effect of MTBP knockdown (MTBP siRNA) in MHCC97-H cells on the on the HGF-induced recruitment of ETS-1 to its downstream gene mmp1’s promoter region. *P < 0.05.

To further examine the effect of MTBP on ETS-1, the recruitment of ETS-1 to its downstream gene’s promoter region was examined using ChIP assays. As shown in **Figure 6**, treatment of HGF induced the recruitment of ETS-1 to *mmp1*’s promoter region. Overexpression of MTBP enhances the recruitment of EST-1 to *mmp1*’s promoter region in MHCC97-L cells (**Figure 6A**) induced by HGF, whereas knockdown of MTBP led to a decrease in HGF-induced recruitment of EST-1 to *mmp1*’s promoter region in MHCC97-L cells (**Figure 6B**) induced by HGF. Therefore, MTBP appears to enhance the HGF-induced recruitment of ETS-1 to its downstream gene’s promoter region.

### The specificity of MTBP enhancing the activation of EBS-Luc in HEK293 cells

**Figure 7:**
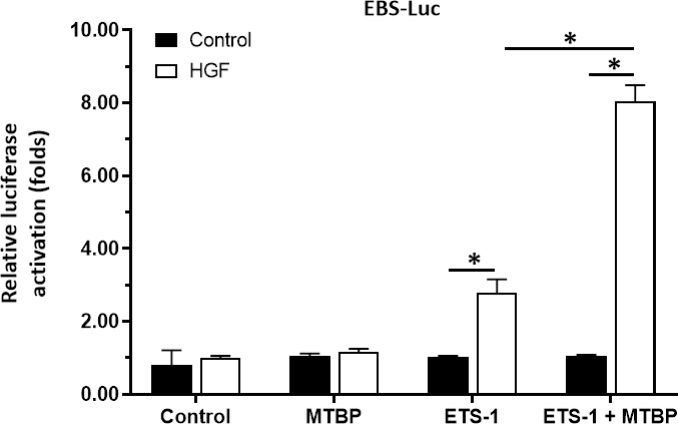
MTBP enhances the activation of EBS-Luc in HEK293 cells. HEK293 cells were cultured and co-transfected with MTBP, siMTBP or ETS-1 with EBS-Luc plasmids and treated with control or HGF. The cells were harvested for luciferase examination. *P < 0.05.

To further confirm the effect and specificity of MTBP on ETS-1’s transcription factor activation, the co-transfection of MTBP, ETS-1 and EBS-Luc in HEK293 cells, a cell line with negative ETS-1 and MTBP expression, was performed. As shown in **Figure 7**, in HEK293 cells, MTBP alone did not affect the activity of EBS-Luc; transfection of ETS-1 could induce the activity of EBS-Luc in the presence of HGF; transfection of MTBP could significantly up-regulate the effect of ETS-1 on the activity of EBS-Luc in the presence of HGF. Therefore, MTBP enhances the activation of EBS-Luc by targeting ETS-1 in the presence of HGF.

### MTBP promotes the *in vitro* proliferation, invasion or migration of HCC cells

**Figure 8:**
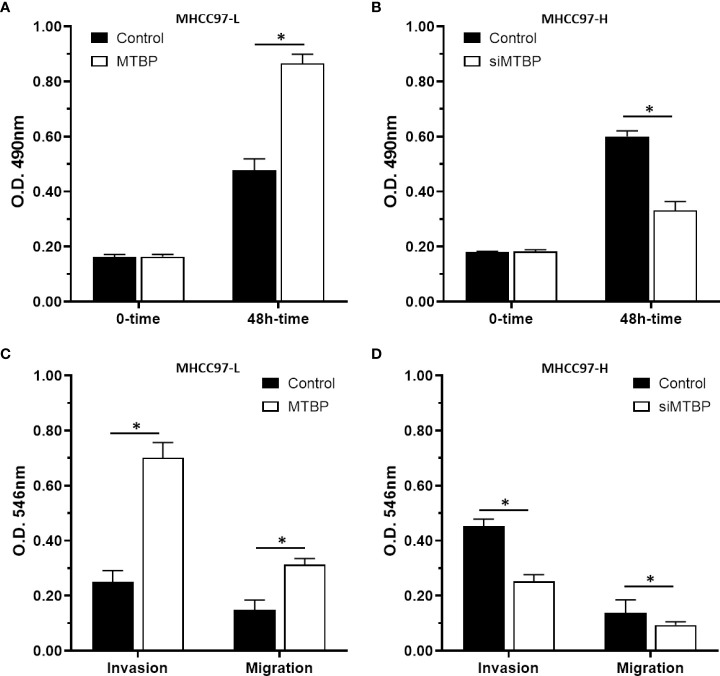
MTBP promotes the *in vitro* proliferation, invasion or migration of HCC cells. **(A–C)** MHCC97-L cells were transfected with control or MTBP expression vectors, and **(B–D)** MHCC97-H cells were transfected with control or siMTBP. The cells were analyzed by MTT**(A, B)** or transwell assays **(C, D)**. *P < 0.05.

The above results indicated that MTBP enhances the activation of ETS-1 which is an important regulator of cellular proliferation or invasion. To further reveal the roles of MTBP, the effect of MTBP on the proliferation, invasion or migration on HCC cells was examined. As shown in **Figure 8**, overexpression of MTBP in MHCC97-L significantly enhanced the proliferation of MHCC97-L cells (**Figure 8A**); whereas knockdown of MTBP in MHCC97-H cells decreased the proliferation of MHCC97-H cells (**Figure 8B**). Similar results were obtained from the transwell experiments: overexpression of MTBP in MHCC97-L significantly enhanced the *in vitro* invasion or migration of MHCC97-L cells (**Figure 8C**); whereas knockdown of MTBP in MHCC97-H cells decreased the *in vitro* invasion or migration of MHCC97-H cells (**Figure 8D**). However, the effect of MTBP on HCC cell invasion is much higher than its effect on HCC cell migration.

### MTBP can upregulate the activity of ETS-1 in tumor tissue

**Figure 9:**
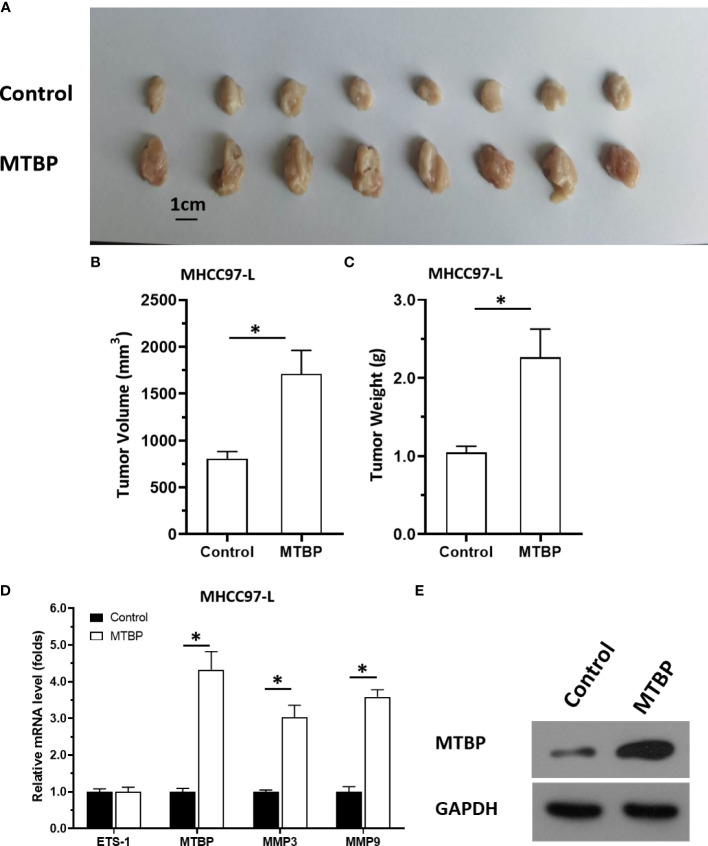
Overexpression of MTBP enhances *in vivo* growth and ETS-1 activation in HCC tumor tissues. MTBP was overexpressed in MHCC97-L cells *via* stable transfection, and cells were injected into nude mice to form subcutaneous tumor tissues. **(A)** the tumor tissues formed by MHCC97-L cells overexpressed MTBP. **(B, C)** the volumes or weights of tumor tissues formed by MHCC97-L cells overexpressed MTBP. **(D)** the expression level of ETS-1, MTBP, MMP3 or MMP9 in the tumor tissues. **(E)** the images of western blot was shown to indicate the stable transfection of MTBP. *P < 0.05.

**Figure 10:**
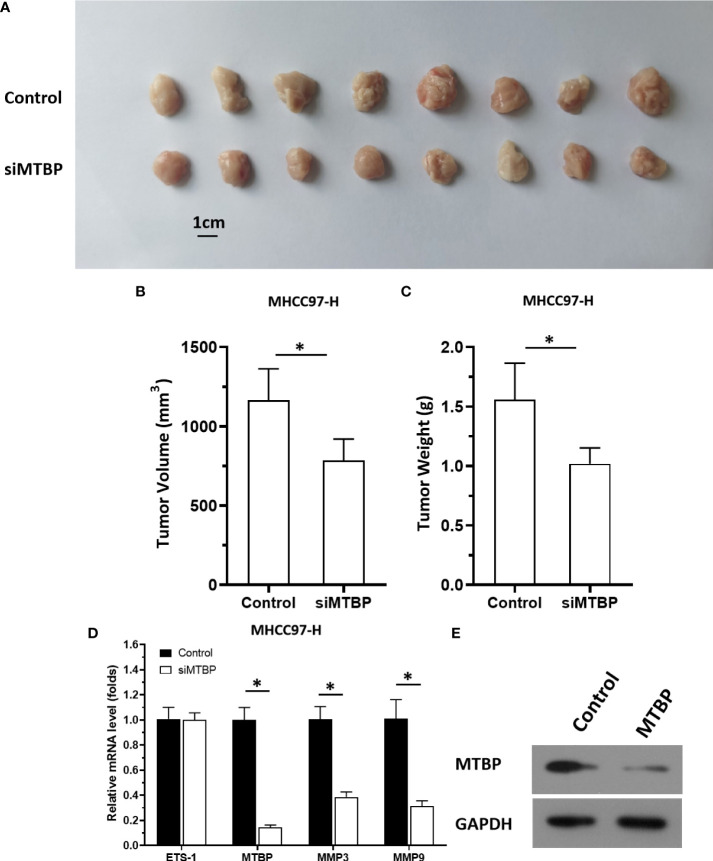
Knockdown of MTBP *via* its siRNA causes a decrease in *in vivo* growth and ETS-1 activation in HCC tumor tissues. The MTBP was knockdown in MHCC97-H cells *via* its siRNA *via* stable transfection and cells were injected into nude mice to form subcutaneous tumor tissues. **(A)** the tumor tissues formed by MHCC97-H cells knockdown MTBP *via* MTBP siRNA. **(B, C)** the volumes or weights of tumor tissues formed by MHCC97-L cells’ knockdown MTBP *via* MTBP siRNA. **(D)** Expression levels of ETS-1, MTBP, mmp3, ormmp9 in the tumor tissues. **(E)** the images of western blot was shown to indicate the stable transfection of siMTBP. *P < 0.05.

**Figure 11:**
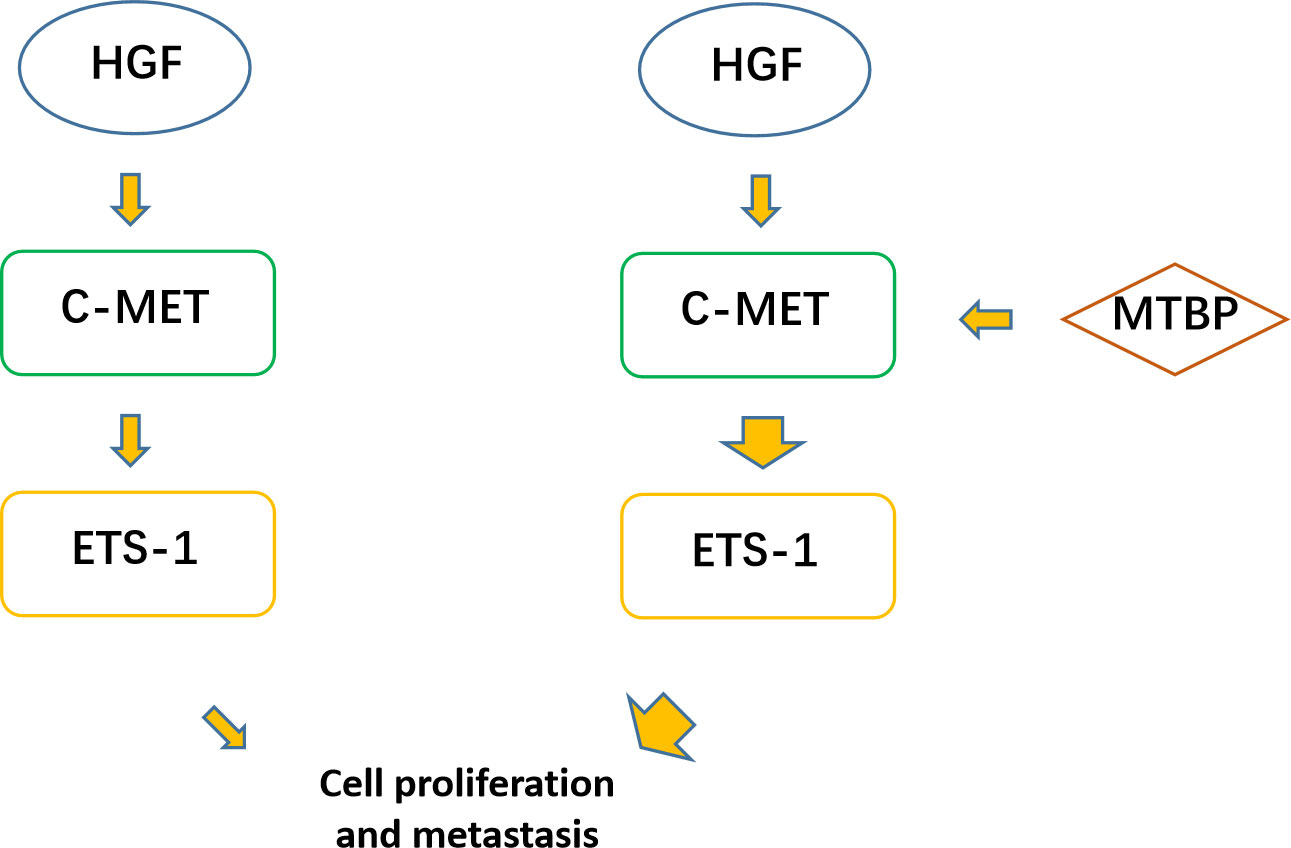
MTBP effects on ETS-1. ETS-1 is a downstream effector (transcription factor) of HGF/c-MET, and HGF can finally activate ETS-1 through activation of c-MET. ETS-1 can promote the proliferation of malignant tumor cells such as HCC by mediating the expression of its downstream genes. MTBP can act as a transcriptional activation cofactor (coactivator) of ETS-1, upregulate the activity of HGF/c-MET/ETS-1 pathway, and ultimately promote the proliferation of HCC cells.

The aforementioned results were all obtained in cultured cells and were further tested in subcutaneous tumor tissues of nude mice. The results are shown in **Figure 9**, overexpression of MTBP in MHCC97-L cells was found not only to enhance the subcutaneous growth of MHCC97-L cells in nude mice, but also up-regulate the activity of ETS-1 and promote a higher expression levels of ETS-1 downstream genes. However, using siRNA to knock down MTBP in MHCC97-H cells could inhibit the tumorigenesis of MHCC97-H cells in nude mice and lead to downregulation of the activity of ETS-1 in the cells and the expression levels of ETS-1’s downstream genes (**Figure 10**). This finding indicates that MTBP could act as a novel co-activator (**Figure 11**) of ETS-1 to promote the proliferative activity of HCC cells *in vivo* and *in vitro.*

### The effect of MTBP on ETS-1 in Huh-7 cells

**Figure 12:**
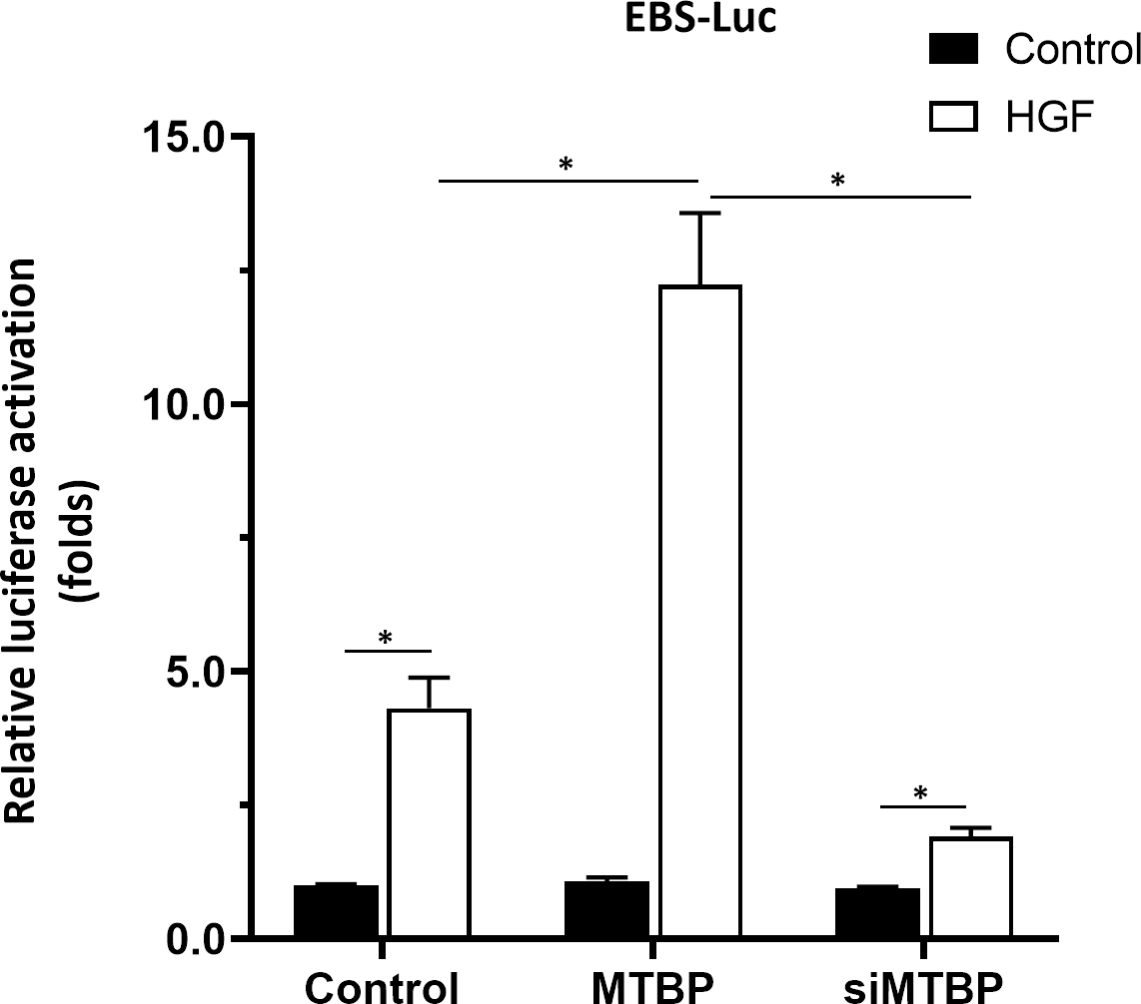
MTBP enhances the activation of EBS-Luc in Huh-7 cells. Huh-7 cells were transfected with control, MTBP or siMTBP and co-transfected with EBS-Luc. Then, cell were analyzed by luciferase. *P < 0.05.

**Figure 13:**
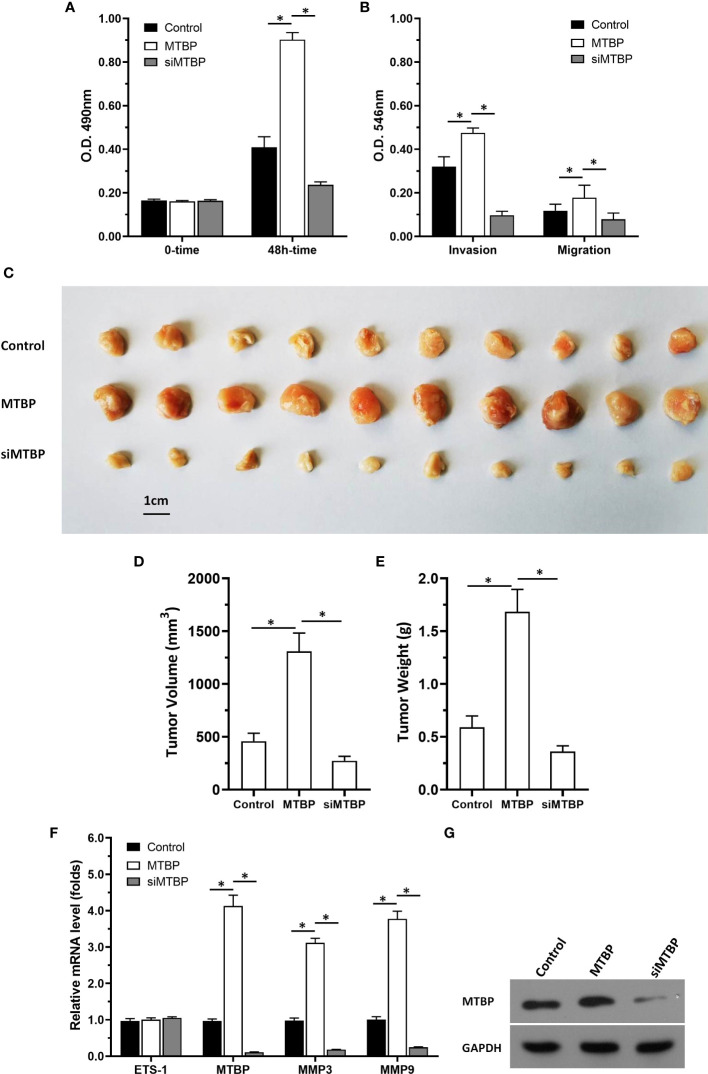
MTBP promotes the *in vitro* or *in vivo* proliferation of Huh-7 cells. Huh-7 cells were stably transfected with control, MTBP or siMTBP. **(A)** Huh-7 cells were analyzed by MTT. **(B)** Huh-7 cells were analyzed by the transwell. **(C–G)** Huh-7 cells were injected into the nude mice to form the subcutaneous tumor model. The results were shown as images of the tumors **(C)**, tumor volumes **(D)**, tumor weights **(E)**, the mRNA level of MTBP, ETS-1, mmp3, mmp9 in tumor tissues *via* qPCR. **(F)** the images of western blot to indicated the stable transfection of MTBP or siMTBP in Huh-7 cells. *P < 0.05.

To further confirm the specificity of MTBP on ETS-1, Huh-7 cell, a P53 deficient HCC cell line was used. As shown in **Figure 12**, overexpression of MTBP enhanced the activation of EBS-Luc in Huh-7 cells and knockdown of MTBP decreased the activation of EBS-Luc, in the presence of HGF. Moreover, the effect of MTBP on Huh-7 cells proliferation, *in vitro* invasion or *in vitro* migration was also examined. As shown in **Figure 13**, overexpression of MTBP enhanced the proliferation (**Figure 13A**), *in vitro* invasion or *in vitro* migration of Huh-7 cell (**Figure 13B**); whereas knockdown of MTBP repressed the proliferation, *in vitro* invasion or *in vitro* migration of Huh-7 cells.

Next, the effect of MTBP on the *in vivo* proliferation of Huh-7 cells or the ETS-1 pathway in tumor tissues was examined by using the subcutaneous tumor models. As shown in **Figures 13C-G**, overexpression of MTBP enhanced the subcutaneous growth of Huh-7 cells in nude mice and knockdown of MTBP repressed the subcutaneous growth of Huh-7 cells. Moreover, MTBP also up-regulated the activity of ETS-1 (enhanced the expression of ETS-1’s downstream genes, *mmp3* or *mmp9*) and using siRNA to knock down MTBP lead to downregulation of the activity of ETS-1 (decreased the expression of ETS-1’s downstream genes, *mmp3* or *mmp9*) (**Figure 13F**). Therefore, MTBP could act as a novel co-activator of ETS-1 to promote the proliferative activity of HCC cells *in vivo* and *in vitro* and did not related to P53/MDM2.

## Discussion

HCC seriously endangers people’s physical and mental health, and its clinical diagnosis, treatment, and related issues have always been the focus of research. At present, it is generally believed that the main risk factor for the occurrence and progression of HCC is the inflammation caused by long-term infections, such as HBV and HCV (46, 47). Under repeated stimulation caused by such inflammation, overexpression of vascular endothelial growth factor receptor (VEGFR) in hepatocytes and continuous and abnormal activation of related pathways, HCC is finally induced, ultimately causing the occurrence and progression of HCC cells (48, 49). This process makes VRGFR and other key factors of cell survival, proliferation, metastasis and invasion, especially tumor angiogenesis, the main intervention targets for HCC anti-tumor therapy (50, 51). However, the problem with targeting VEGFR and other therapeutic sites is that inhibiting a single signaling pathway may have anti-tumor activity that can be easily compensated by other signaling pathways (52–55). To solve this problem, the current anti-tumor drug treatment strategies for HCC are mainly molecularly-targeted drugs such as various multi-targeted tyrosine kinase inhibitors (TKIs) (56–58). Although the existing multi-target TKIs, such as Sorafenib, Regorafenib, Lenvatinib and Cabozantinib can target on multiple pathways to avoid compensation between signaling pathways to a certain extent, the efficacy of these drugs are still unsatisfactory (59). Moreover, the toxic and side effects of these TKIs could not be ignore (60–63). Patients with advanced HCC are often accompanied by different degrees of liver fibrosis or liver cirrhosis (64, 65). Portal hypertensive gastrointestinal disease caused by liver fibrosis or liver cirrhosis not only induces injuries of gastrointestinal digestive system; but also affects the absorption of TKIs and significantly increased risk of gastrointestinal bleeding in patients receiving TKIs (66–69). At the same time, TKIs can damage the function and regeneration of the mucosa and epithelium of the digestive tract, causing serious toxic side effects by targeting the RTKs represented by VEGFR which is essential for the integrity and cellular regeneration of mucosal epithelium (70, 71). Therefore, the need for research and development of new therapeutic targets is present.

Transcription factor ETS-1 is an important regulator of cell proliferation, survival, metastasis, and invasion, and its activity is mainly affected by upstream cascade HGF/c-MET and can be regulated by AKT or MAPK (56–58, 72). In addition to this step, the transcription factor activity of ETS-1 is mainly affected by various transcriptional regulators/cofactors (73, 74). In this study, MTBP can act as a novel co-activator of ETS-1 and lead to upregulation of the transcription factor activity of ETS-1. MTBP was first identified as a regulator of MDM2 (the MDM2-interacting protein) (16–20). Some recent evidence has revealed that MTBP can function as a pro-proliferative and oncogenic regulator in human malignancies by some potential mechanisms (16–20): MTBP may promote the proliferation of cancer cells by function as a co-amplified of c-MYC, which is a typical oncogene, or ZEB2, one of the key regulators of the epithelial–mesenchymal transition (EMT) process (16–20, 75–77). Based on the evidence that MTBP is an MDM2 interaction protein, MTBP could also inhibit apoptosis *via* suppression of the MDM2/P53 pathway and promotion of cancer cell metastasis through the MDM2-mediated the degradation of E-Cadherin, a typical indicator of epithelial features (78). EMT is an important physiological process of tumor cells, which can reduce cell polarity and is closely related to cell proliferation, metastasis, invasion, and drug resistance 54–87). These existing results suggest that MTBP is closely related to cellular EMT. Based on these studies, the results of this study further expand our understanding of MTBP: MTBP functions as a novel co-activator of ETS-1. This finding is reflected in MTBP interaction with ETS-1 and enhancement of the transcription factor activation of ETS-1. In this study, the transcription factor activity of ETS-1 was detected by luciferase and qPCR assay, and the recruitment of ETS-1 in the promoter region of its downstream gene mmp1 could also directly reflect the effects of MTBP on ETS-1. The role of ETS-1 in HCC has been widely reported (88, 89). It is worth mentioning that the inhibitor of ETS-1 used in this study was ARQ-197, which is an inhibitor of c-MET (88, 89). A report by Jie et al. also showed that small molecular inhibitors of ETS-1 can effectively achieve antitumor activity in HCC cells (90). Further studies using inhibitors of ETS-1 will be conducted in the future (90). The results of Shao et al. showed that ETS-1 could act as a co-activator of PXR to upregulate the activity of PXR and induce drug resistance in HCC cells (91). Our previous results also showed that MTBP could act as a co-activator of PXR (21). Therefore, in the future, whether MTBP is associated with ETS-1 and PXR form a ternary complex will be further explored.

It is worth mentioning that, the function of MTBP is closely related to MDM2 and P53 (P53/MDM2 pathway) (92, 93), but MDM2 and P53 are not the only molecular mechanisms of MTBP, and MTBP can also function *via* other proteins (such as the interaction between MTBP and PXR in the previous results of our group; the interaction of MTBP with ETS-1 in the presence work). To this end, the overexpression or knockdown of MTBP in a P53-deficient cell line, Huh-7 cells, was examined. Our results showed that MTBP enhanced the activation of ETS-1 in Huh-7. This further confirms the specificity of the action of MTBP: its effect on ETS-1 is independent of the P53/MDM2 pathway. Meanwhile, since ETS-1 is a key regulator of cell proliferation, metastasis, and invasion, our results used MTT, Transwell, and nude mouse tumorigenic models to examine the effects of MTBP on HCC cell proliferation, invasion, and metastasis, respectively. The results showed that MTBP could promote the proliferation, metastasis and invasion of three HCC cells. Interestingly, the effects of MTBP overexpression or knockdown on the *in vitro* invasion of HCC cells greatly outweighed the effects on *in vitro* migration of HCC cells. The reason may be that the main mechanism of ETS-1 is to induce the expression of matrix metalloproteinases, and then destroy the tissue matrix through mmps, and finally induce the invasion of HCC and other cancerous cells (94–97). However, ETS-1 is a transcription factor with a wide range of functions, and MTBP can also promote the proliferation and *in vitro* migration of HCC cells through ETS-1. It is worth mentioning that MTBP can up-regulate the expression levels of ETS-1 downstream genes in HCC tumor tissue.

Finally, to summarize the main points of this paper, we reported and elucidated the interaction between MTBP and ETS-1 and its clinical significance for the first time in HCC cells, which not only expands our understanding of MTBP and ETS-1, but also provides HCC-related research provides more insights. At the same time, we also highlight the areas that can be improved in the future: (1) It is necessary to locate the interaction between MTBP and ETS-1, and try to determine the key amino acid residues of the interaction between the two; (2) Try to develop small molecules of MTBP Inhibitors; (3) Attempt to establish transgenic animals

## Data availability statement

The original contributions presented in the study are included in the article/supplementary material. Further inquiries can be directed to the corresponding author.

## Ethics statement

The studies involving human participants were reviewed and approved by the ethics committee of the Fifth Medical Center, General Hospital of Chinese PLA (People’s Liberation Army). The patients/participants provided their written informed consent to participate in this study. The animal study was reviewed and approved by the Animal Ethics Committee of the Fifth Medical Center of the Chinese People’s Liberation Army General Hospital.

## Author contributions

B-AL, HW, and FC: concept, design, statistics, data collection, manuscript writing, final approval. LZ, QB, LL, YZ: design, statistics, data collection. ZX and XN: concept, data collection. DZ and HX: statistics, data collection. All co-authors contributed to the article and approved the submitted version.

## Conflict of interest

The authors declare that the research was conducted in the absence of any commercial or financial relationships that could be construed as a potential conflict of interest.

## Publisher’s note

All claims expressed in this article are solely those of the authors and do not necessarily represent those of their affiliated organizations, or those of the publisher, the editors and the reviewers. Any product that may be evaluated in this article, or claim that may be made by its manufacturer, is not guaranteed or endorsed by the publisher.
